# Leveraging homologous recombination deficiency for sarcoma: Unravelling homologous recombination repair deficiency and therapeutic opportunities in soft tissue and bone sarcoma

**DOI:** 10.1007/s00292-024-01381-y

**Published:** 2024-11-13

**Authors:** Lara Planas-Paz, Chantal Pauli

**Affiliations:** 1 Department of Pathology and Molecular Pathology, University Hospital Zurich, Zurich, Switzerland; 2 Medical Faculty University of Zurich, Zurich, Switzerland

**Keywords:** Precision oncology, Combination therapy, DNA damage response, Cancer organoids, PARPi, Präzisionsonkologie, Kombinationstherapie, DNA-Schadensreaktion, Krebsorganoide, PARPi

## Abstract

**Background:**

Homologous recombination deficiency (HRD) in tumors correlates with poor prognosis and metastases development. Determining HRD is of major clinical relevance as it can be treated with PARP inhibitors (PARPi). HRD remains poorly investigated in sarcoma, a rare and heterogeneous cancer of mesenchymal origin.

**Objective:**

We aimed (i) to investigate predictive biomarkers of HRD in several independent sarcoma cohorts using a cross-functional strategy by combining genomic, transcriptomic and phenotypic approaches and (ii) to evaluate the therapeutic potential of PARPi and DNA damage response (DDR)-based therapies *ex vivo*.

**Materials and methods:**

We performed a comprehensive genomic and transcriptomic characterization of sarcoma using datasets from The Cancer Genome Atlas (TCGA) and Therapeutically Applicable Research to Generate Effective Treatments (TARGET), and our own bone and soft tissue sarcoma cohorts. We evaluated PARP1/2 and WEE1 inhibition *ex vivo *in patient-derived sarcoma cell models as monotherapy and in combination with chemotherapeutic agents to identify synergistic effects.

**Results:**

Firstly, we identified genomic traits of HRD in a subset of sarcomas associated with molecular alterations in homologous recombination repair (HRR) pathway genes and high chromosomal instability. Secondly, we identified and validated distinct SARC-HRD transcriptional signatures that predicted sensitivity to PARPi. Finally, we showed functional defects in HRR in sarcoma cells that were associated with functional dependency towards PARPi and WEE1i and support the clinical use of RAD51 as a predictive biomarker for PARPi sensitivity.

**Conclusion:**

We provide a personalized oncological approach to potentially improve the treatment of sarcoma patients. We encourage the evaluation of gene expression signatures to enhance the identification of patients who might benefit from DDR-based therapies.

## Abstract (de)

**Hintergrund:**

Homologe Rekombinationsdefizienz (HRD) in Tumoren korreliert mit einer schlechten Prognose und der Entwicklung von Metastasen. Die Bestimmung der HRD ist von großer klinischer Relevanz, da sie mit Inhibitoren der Poly(ADP-Ribose)-Polymerase (PARPi) behandelt werden kann. Dies erfolgte bislang insbesondere bei Ovarial‑, Brust- und Prostatakrebs. Bei Sarkomen, einem seltenen und heterogenen Krebs mesenchymalen Ursprungs, ist die HRD noch wenig untersucht.

**Ziel der Arbeit:**

Unser Ziel war es, (i) prädiktive Biomarker für HRD in Sarkomen mithilfe molekularer und phänotypischer Daten zu untersuchen und (ii) das therapeutische Potenzial von PARPi bei Sarkomen mit HRD und DDR-basierter Therapien („DNA damage response“) *ex vivo *funktional zu testen.

**Material und Methoden:**

Dazu wurde eine umfassende molekulare Charakterisierung mittels genomischer und transkriptomischer Daten von Sarkomen aus TCGA, TARGET und unserer eigenen Datenbank durchgeführt. Wir untersuchten die PARPi- und WEE1-Medikamentensensitivität in neu etablierten Sarkomzellmodellen mit HRD. Untersucht wurden die Monotherapie sowie die Kombinationstherapie mit Chemotherapeutika, um synergistische Effekte zu identifizieren.

**Ergebnisse:**

Mittels einer breiten molekularen Analyse konnten wir HRD-Merkmale in unterschiedlichen Sarkomsubgruppen identifizieren. Die molekularen Veränderungen in den Homologe-Rekombinationsreparatur(HRR)-Signalwegen zeigen eine hohe Korrelation mit der chromosomalen Instabilität der individuellen Sarkome. Mittels eines mathematischen Models wurden die Sarkome in HRD+ und HRD− gruppiert. Aufgrund dieser Einteilung konnten wir eine prädiktive SARC-HRD-Transkriptionssignatur identifizieren und funktional validieren, welche die Empfindlichkeit gegenüber PARP- und WEE1-Inhbitoren vorhersagt und den klinischen Einsatz von RAD51 als prädiktiven Biomarker für PARPi-Sensitivität unterstützt.

**Schlussfolgerung:**

In der vorliegenden Arbeit wird ein personalisierter onkologischer Ansatz zur Verbesserung der Behandlung von Sarkompatient:Innen beschrieben. Patient:Innen mit HRD+-Sarkomen können von Therapien mit Medikamenten, welche im DNA-Reparatursystem angreifen, profitieren.

## Introduction

Defects in homologous recombination repair (HRR) are common in several cancer types but remain poorly understood in sarcoma, a rare and heterogeneous cancer of mesenchymal origin [1, 8]. Determining HRR deficiency is of high clinical relevance as it is associated with susceptibility to poly (ADP-ribose) polymerase (PARP) inhibition [11, 13]. Standard-of-care treatment for advanced-disease sarcoma mostly relies on chemotherapy. Unfortunately, patients often show chemoresistance, and metastatic disease is associated with poor survival [1, 12]. Therefore, identifying underlying disease mechanisms for the distinct sarcoma entities is likely to unlock new therapeutic avenues.

## Molecular characterization of genomic instability signatures in sarcoma

Homologous recombination deficiency (HRD) is frequently observed in ovarian and breast cancer, followed by prostate and pancreatic cancer. Accurate detection of HRD is of clinical relevance as it is indicative of sensitivity to targeted therapy with PARP inhibitors (PARPi) and DNA-damaging agents [13]. The most clinically widespread genetic biomarker of HRD is germline or somatic *BRCA1/2 *mutation status, hereafter termed BRCA*ness *[8]. However, the prevalence of HRD extends beyond *BRCA1/2 *inactivation [11]; therefore, the term HRD*ness* will be used throughout this article to encompass tumor-agnostic HRR pathway deficiencies.

**Fig. 1:**
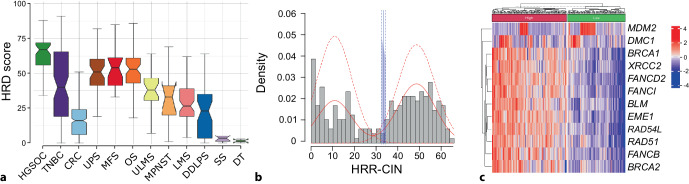
**a** Homologous recombination deficiency (*HRD*) score in nine different sarcoma entities compared with high-grade serous ovarian cancer (*HGSOC*), triple-negative breast cancer (*TNBC*), and colorectal cancer (*CRC*). **b** Histogram of HRR-CIN in soft tissue sarcoma showing a bimodal distribution and the optimal cut-off value for the HRD score. **c** Heatmap with hierarchical clustering of 10 differentially expressed HRR genes, named SARC-HRD signature, in HRD^high^ compared with HRD^low^ sarcoma cases. *UPS* undifferentiated pleomorphic sarcoma, *MFS* myxofibrosarcoma, *OS* osteosarcoma, *ULMS* uterine leiomyosarcoma, *LMS* extra-uterine leiomyosarcoma, *DDLPS* dedifferentiated liposarcoma, *SS* synovial sarcoma, *DT* desmoid tumor. Data information: datasets from TCGA-SARC (*n* = 247), TCGA-OV (*n* = 61), TCGA-BRCA (*n* = 92), TCGA-COAD (*n* = 385), and TARGET-OS (*n* = 69) were used; *n* indicates biological replicates. Data in **a** are median ± third and first quartile, the *whiskers* are minimum and maximum values

To identify possible genomic traits of HRD*ness* in sarcoma, we analyzed signatures of genomic instability in the TCGA cohort of 247 soft tissue sarcoma (STS) cases and the TARGET cohort of 69 osteosarcoma cases [1]. For each individual case, we computed loss-of-heterozygosity (LOH), large-scale transitions (LST), and telomeric allelic imbalances (TAI) as well as the HRD score as the unweighted sum of all three values [9]. The HRD score is a clinically validated biomarker that predicts PARPi and platinum sensitivity in high-grade serous ovarian and triple-negative breast carcinoma (HGSOC and TNBC, respectively) [4, 15], which were used as controls in our analysis. Sarcoma entities known to present high levels of genomic instability, namely myxofibrosarcoma, undifferentiated pleomorphic sarcoma, and osteosarcoma, also exhibited the highest HRD scores, followed by uterine leiomyosarcoma, malignant peripheral nerve sheath tumor, dedifferentiated liposarcoma, and non-uterine leiomyosarcoma. Both synovial sarcoma and desmoid tumors, associated with chromosomal translocations or point mutations in specific genes, presented low HRD scores (Fig. 1a).

A thorough analysis of the molecular landscape of STS identified numerous alterations in HRR pathway genes: 22% of STS patients carried mutations in *BRCA2*, and 37% and 20% in the Fanconi anemia genes *FANCB* and *FANCA*, respectively. The TP53 regulator *MDM2*, the tumor suppressor *PTEN*, cell cycle checkpoint regulators *RAD1 *and *CHEK1*, and the DNA damage and replication proteins *ATM, RPA1*, and *H2AFX* completed the top 10 altered gene list in the TCGA-SARC cohort. As the total number of HRR alterations, referred to as HRR-CIN, followed a bimodal distribution, we applied a finite mixture model that separated the cohort into two subpopulations. The HRD score for each datapoint was associated with the respective HRR-CIN group in a receiver operator characteristic (ROC) curve, and an optimal cut-off value for stratifying patients based on HRD score was determined at 32 (Fig. 1b). Of note, an HRD score above 42 has been clinically validated to predict the response to platinum-containing neoadjuvant chemotherapy in patients with TNBC and recurrent ovarian cancers treated with niraparib [4, 15]. Currently, no clinically validated diagnostic test exists for drug response prediction in soft tissue or bone sarcoma. Besides elevated levels of genomic alterations in STS with high HRD scores (HRD^high^), a high degree of chromosomal instability (CIN), majorly influenced by high levels of chromosomal gains, as well as molecular signatures associated with deficiency in HRR were identified [14]. Altogether, we identified distinct sarcoma entities with multiple traits of HRD*ness*, thus building the rationale for PARPi therapy.

## Transcriptional signature of homologous recombination deficiency in sarcoma

To explore the transcriptomic landscape of sarcoma with HRD*ness*, we investigated gene expression profiles and signaling pathway enrichment in HRD^high^ compared with HRD^low^ STS patients from the TCGA cohort. Sarcomas exhibiting HRD*ness* showed a general enrichment of DNA damage repair pathways, including HRR and mismatch and nucleotide excision repair, as well as significant upregulation of 10 key HRR genes: *BRCA1, BRCA2, BLM, EME1, FANCB, FANCD2, FANCI, RAD51, RAD54L*, and *XRCC2*, which we named the SARC-HRD signature (Fig. 1c). Interestingly, these 10 HRR genes were also upregulated in five HRD^high^ compared with five HRD^low^ patient-derived *ex vivo* sarcoma cell models, concomitantly with increased genomic instability traits, such as high CIN and an elevated number of genomic alterations in HRR genes. Our combined results show that STSs harbor numerous genomic traits of HRD*ness* that are shared with transcriptional upregulation of a distinct gene expression signature.

## DDR-targeting opportunities for sarcoma with HRD*ness* traits

**Fig. 2:**
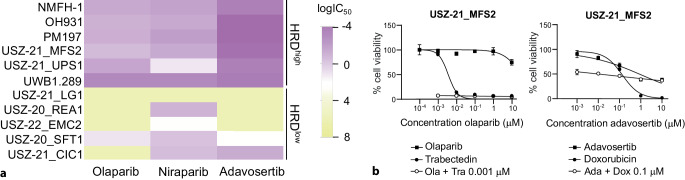
**a** Heatmap of the half-maximal inhibitory concentration (IC_50_) showing sensitivity to PARPi and WEE1i in HRD^high^ but not HRD^low^ sarcoma cell models. The ovarian carcinoma cell line UWB1289 with *BRCA1* mutations was used as a positive control for PARPi response and HRD*ness*. **b** An HRD^high^ myxofibrosarcoma cell model treated for 3 days with five doses of olaparib alone and in combination with 1 nM trabectedin or five doses adavosertib alone and in combination with 100 nM doxorubicin

PARP1 and PARP2 are core players in base-excision-mediated DNA repair (BER). PARPi block the BER pathway and HRR-deficient cells must rely on the error-prone, non-homologous end-joining pathway for double-strand DNA repair. Cells accumulate genetic damage that ultimately induces cell death [11]. *In* *vitro* sensitivity to DNA double-strand break-inducing drugs, such as platinum salts, is also a feature of HRD cells. To investigate whether sarcoma with HRD*ness* traits respond to PARPi and platinum chemotherapy, we explored the sensitivity of five HRD^high^ and five HRD^low^ patient-derived *ex vivo* sarcoma cell models to two PARPi (olaparib and niraparib), a platinum-based antineoplastic drug (oxaliplatin), and standard chemotherapy for STS patients (doxorubicin and trabectedin). We evaluated drug response using an ATP-based viability assay after cell treatment with six drug doses. We used a *BRCA1*-mutated ovarian cancer cell line as a positive control for PARPi response. Similarly to *BRCA1*-mutated ovarian cancer cells, HRD^high^ sarcoma cells showed higher sensitivity than HRD^low^ sarcoma cells to PARPi but no major differential response to chemotherapy (Fig. 2a). PARPi are tested in sarcoma clinical trials in combination with standard chemotherapy, and we thus assessed potential synergism between olaparib and trabectedin in our ex vivo sarcoma cell models [5]. The combinatorial modality resulted in synergistic effects in HRD^high^ sarcoma cells as well as in *BRCA1*-mutated ovarian cancer cells but not in HRD^low^ sarcoma cells (Fig. 2b).

We hypothesized that inhibitors targeting other DDR mechanisms might also exhibit HRD-dependent synthetic lethality in sarcoma cells. We focused on the serine/threonine kinase WEE1, known to inhibit CDK1 and CDK2, thus blocking G2/M progression and promoting DNA repair [10]. To analyze the effect of WEE1 inhibition on sarcoma cell viability, we subjected five HRD^high^ and five HRD^low^ patient-derived *ex vivo* sarcoma cell models to the WEE1 inhibitor (WEE1i) adavosertib. Adavosertib elicited a dose-dependent decrease in cell viability in HRD^high^ sarcoma cells (Fig. 2a). Moreover, combination of adavosertib and doxorubicin showed synergistic effects in HRD^high^ but not in HRD^low^ sarcoma cells (Fig. 2b). Altogether, our data show that targeting the DNA damage response and DNA repair pathways are powerful therapeutic strategies for sarcoma with HRD*ness* traits.

## RAD51 nuclear foci formation as a marker of deficient homologous recombination repair in sarcoma

**Fig. 3:**
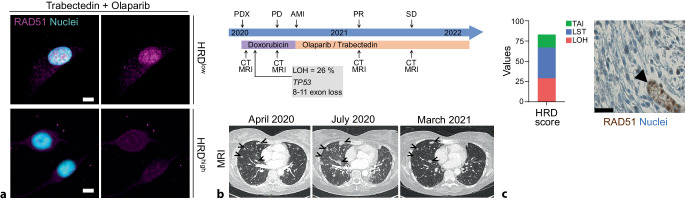
**a** Immunofluorescence showing RAD51 nuclear foci (magenta) upon 6 h treatment with 10 nM trabectedin and 100 nM olaparib in combination only in the HRD^low^ sarcoma cell model. **b** Timeline of LMS patient diagnosis and treatment. Magnetic resonance imaging (*MRI*) of the LMS patient since primary diagnosis. Open arrowheads point at metastatic lesions. *PDX* primary diagnosis, *PD* progressive disease, *AMI* acute myocardial infarction, *PR* partial response, *SD* stable disease, *CT* computer tomography, *MRI* magnetic resonance imaging. **c** RAD51 IHC in the metastatic patient’s tissue. Compare RAD51 nuclear expression in normal tissue (arrowhead points at gastric gland) but not in tumorous gastric tissue. Scale bars 10 µm (**a**) and 25 µm (**c**)

RAD51 plays a crucial role in DNA replication and HRR, catalyzing the recognition of homology and strand exchange between partner DNA strands, one with a processed DNA break and the other acting as the repair template [7]. The formation of RAD51 nuclear foci is regarded as a functional biomarker of HRR proficiency and can be predictive of PARPi resistance [2, 3, 6]. To functionally assess HRR in sarcoma cell models *ex vivo*, we evaluated the formation of RAD51 nuclear foci after eliciting DNA damage with olaparib and trabectedin, either as monotherapy or in combination. Drug-induced DNA damage was evaluated by immunostaining for the phosphorylated form of the histone variant H2A.X (γH2A.X). Higher γH2A.X expression was measured in cells treated with the combination regimen compared with the single agents, regardless of their HRD status. Notably, while HRD^low^ sarcoma cells formed RAD51 nuclear foci upon olaparib- and trabectedin-induced DNA damage, neither formation of RAD51 nuclear foci nor increased RAD51 nuclear intensity were observed in HRD^high^ sarcoma cells and *BRCA1*-mutated ovarian cancer cells (Fig. 3a). Patient tissue samples from which the HRD^high^ cells were derived also showed reduced RAD51 expression and nuclear foci. Altogether, our combined results show deficiency in HRR in PARPi-sensitive patient-derived sarcoma cells.

## Clinical translation and therapeutic opportunities for sarcoma patients

A 47-year-old female patient presenting with multiple lesions in the lung and liver was diagnosed in April 2020 with metastatic leiomyosarcoma, most likely of uterine origin. The patient underwent SOC and was treated with pegylated liposomal doxorubicin (PLD). Broad molecular profiling revealed *TP53* loss, stable microsatellite status, tumor mutational burden of 7 mut/mb, and an LOH score of 26%, considered high in the hospital-based molecular tumor board. Due to an anthracycline-induced cardiomyopathy, PLD treatment was terminated, and the patient underwent off-label treatment with trabectedin and olaparib, remaining in a lasting radiological partial remission for one and a half years (Fig. 3b). Whole-genome sequencing of a stomach metastasis in late 2022 revealed an HRD score of 83 and genomic alterations in multiple HRR genes. Moreover, the absence of nuclear RAD51 expression in the metastatic tissue further evidenced defective HRR (Fig. 3c). This case highlights the clinical benefit of extending the therapeutic indications for PARPi alone or in combination with standard chemotherapy.

The efficacy of both olaparib and trabectedin combination therapy as well as of adavosertib in combination with chemotherapeutic agents is currently under clinical investigation in either metastatic or advanced sarcoma (NCT04076579), recurrent ovarian cancer (NCT02101775), or relapsed or refractory solid tumors in pediatric patients (NCT02095132). Results from non-biomarker- and HRD score-guided clinical trials will likely evidence the clinical benefit of DDR-targeting agents for sarcoma patients. In addition, the incorporation of transcriptomic profiling in clinical practice holds significant biomarker potential as gene expression changes faithfully reflect the rapidly changing molecular dynamics of the cell. Future research will determine whether incorporating transcriptomic biomarkers into clinical trial design can improve the clinical management of STS patients.

## Practical conclusion

- Distinct sarcoma entities in multiple patient cohorts harbor genomic traits of HRD*ness*, such as elevated genomic instability signatures, numerous alterations in homologous recombination repair (HRR) genes and chromosomal instability; routine evaluation of such features in sarcoma patients may prove beneficial to guide individualized patient treatment.
- At the transcriptomic level, identification of the SARC-HRD gene expression signature as predictive of PARPi sensitivity warrants further clinical validation; incorporating transcriptomic profiling may offer significant advances in patient stratification and PARPi response prediction.
- Both patient-derived sarcoma cell models and a sarcoma patient with HRD*ness* responded to a combinatorial regimen of chemotherapeutic and DNA damage response-targeting agents, thus suggesting a widespread therapeutic benefit of such combinations.
- Future research could benefit from exploring specific mechanisms of PARPi resistance in sarcoma and focus on the identification of treatment combination regimens to prevent and overcome development of resistance.

## Declarations

The present study was conducted following regional/cantonal and institutional guidelines and in compliance with the WMA Declaration of Helsinki and after approval by our cantonal ethical review board Zurich (BASEC-2021-00417). All studies mentioned were in accordance with the ethical standards indicated in each case.

The supplement containing this article is not sponsored by industry.
